# Immune-related prognosis biomarkers associated with osteosarcoma microenvironment

**DOI:** 10.1186/s12935-020-1165-7

**Published:** 2020-03-16

**Authors:** Weifeng Hong, Hong Yuan, Yujun Gu, Mouyuan Liu, Yayun Ji, Zifang Huang, Junlin Yang, Liheng Ma

**Affiliations:** 1 Department of Medical Imaging, The First Affiliated Hospital of Guangdong Pharmaceutical University, No.19, Nong Lin Xia Road, Yuexiu District, Guangzhou, 510030 Guangdong China; 2 Department of Oncology, Ruijin Hospital, Shanghai Jiao Tong University School of Medicine, Shanghai, China; 3 The First Affiliated Hospital of Sun Yat-sen University, No.58, Zhongshan Second Road, Yuexiu District, Guangzhou, 510030 Guangdong China; 4 Morning Star Academic Cooperation, Shanghai, China

**Keywords:** Osteosarcoma, Immune-related genes, Microenvironment

## Abstract

**Background:**

Osteosarcoma is a highly aggressive bone tumor that most commonly affects children and adolescents. Treatment and outcomes for osteosarcoma have remained unchanged over the past 30 years. The relationship between osteosarcoma and the immune microenvironment may represent a key to its undoing.

**Methods:**

We calculated the immune and stromal scores of osteosarcoma cases from the Target database using the ESTIMATE algorithm. Then we used the CIBERSORT algorithm to explore the tumor microenvironment and analyze immune infiltration of osteosarcoma. Differentially expressed genes (DEGs) were identified based on immune scores and stromal scores. Search Tool for the Retrieval of Interacting Genes Database (STRING) was utilized to assess protein–protein interaction (PPI) information, and Molecular Complex Detection (MCODE) plugin was used to screen hub modules of PPI network in Cytoscape. The prognostic value of the gene signature was validated in an independent GSE39058 cohort. Gene set enrichment analysis (GSEA) was performed to study the hub genes in signaling pathways.

**Results:**

From 83 samples of osteosarcoma obtained from the Target dataset, 137 DEGs were identified, including 134 upregulated genes and three downregulated genes. Functional enrichment analysis and PPI networks demonstrated that these genes were mainly involved in neutrophil degranulation and neutrophil activation involved in immune response, and participated in neuroactive ligand–receptor interaction and staphylococcus aureus infection.

**Conclusions:**

Our study established an immune-related gene signature to predict outcomes of osteosarcoma, which may be important targets for individual treatment.

## Background

Osteosarcoma is the primary malignant bone cancer that most commonly affects children, adolescents, and young adults [1]. For patients who have metastatic disease at diagnosis or who relapse, the 5-year survival rate is below 30% [2]. Osteosarcoma exhibits a predilection to occur in the metaphysis of long bones, and most commonly occurs in the distal femur (43%), proximal tibia (23%), or humerus (10%) [3]. Clinical outcomes and treatment modalities for osteosarcoma have not changed for more than 30 years. EURAMOS-1 study investigating whether intensified postoperative chemotherapy for high-grade patients failed to improve survival [4, 5], underscoring a critical need for new treatment strategies. The high morbidity and mortality burden in osteosarcoma necessitates additional research to characterize and understand the underlying mechanisms [6, 7].

Tumor microenvironment (TME) was the cellular environment including immune cells, mesenchymal cells, endothelial cells, inflammatory mediators and extracellular matrix molecules. The osteosarcoma bone microenvironment consists of osteoclasts, osteoblasts and hematopoietic cells from which monocytes/macrophages derive [8–10]. All of these cells release multiple growth factors and cytokines with contrasting effects. It is widely considered that the microenvironment plays a significant role in tumor development [11]. Therefore, the comprehensive analysis of the correlation between immune-related gene signatures and overall survival may shed light on pathogenesis of osteosarcoma.

In our study, we calculated immune and stromal scores based on the ESTIMATE algorithm to detect the correlation between immune/stromal scores and clinical parameters. We also calculated the percentage of every kind of immune cells according to the CIBERSORT algorithm to explore the relationship between immune score and immune cells. The above two algorithms can be applied to predict the immune microenvironment of osteosarcoma and better understand the immune characteristics of osteosarcoma.

Our study aimed to construct an immune-related gene signature to predict the prognosis of osteosarcoma patients. We found that 137 genes were dysregulated in 83 osteosarcoma samples, including 134 upregulated genes and three downregulated genes. Two hub genes, SIGLEC7 and SP140, were positively associated with outcomes in osteosarcoma patients. Additionally, the prognostic power of the genes was verified in another independent Gene Expression Omnibus (GEO) dataset.

## Methods

### Data preparation

Clinical information of osteosarcoma patients was downloaded from the Target database (https://ocg.cancer.gov/programs/target). For validation, the gene expression profiles of GSE39058 cohort were downloaded from GEO database (https://www.ncbi.nlm.nih.gov/geo/) [12–14].

Immune scores and stromal scores were calculated based on the ESTIMATE algorithm [15]. We divided osteosarcoma patients into high vs. low score groups according to the median value of immune/stromal scores. Based on their scores, we constructed Kaplan–Meier survival curves, and analyzed the association between immune/stromal scores and clinicopathologic parameters.

We calculated the relative percentage of immune cell types in each osteosarcoma sample according to the CIBERSORT algorithm [16]. The proportion of immune cells in high- and low-immune score groups was depicted in the heatmap. We further applied the Wilcox test to compare the differences in microenvironment between high and low immune score groups. Using Spearman correlation analysis, we confirmed the relationship between immune cells and immune/stromal scores. All analyses were carried out by R version 3.5.2.

### Identification of differentially expressed genes (DEGs) and UpSet plot

We divided osteosarcoma samples into high vs. low immune-score and stromal-score groups according to the median value. Data analysis was performed using package limma [17]. Fold change > 1.5 and adj. p < 0.05 were set as the cutoffs to screen for DEGs.

Intersections between different groups of osteosarcoma were investigated by UpSet R [18]. UpSet R is a novel R package which provides intersecting sets using matrix design, along with visualizations of several common sets, element, and attribute related tasks.

### Enrichment analysis of DEGs

Functional enrichment analysis of DEGs was performed by clusterProfiler R [19] to identify Gene ontology (GO) categories by biological processes (BP), molecular functions (MF), or cellular components (CC) [20]. Pathway enrichment analysis of DEGs was also performed by clusterProfiler R. False discovery rate (FDR) < 0.05 was used as the cut-off. Then, the most significant top 30 pathways were chosen for visualization.

### Human protein–protein interaction (PPI) analysis

Human PPIs were deposited in the search tool for the retrieval of interacting genes (STRING) database (http://string-db.org/) [21], from which we performed a comprehensive analysis and annotation of functional interactions of genes. The STRING database integrates multiple databases that provide information sufficient for the prediction of candidate protein interaction relationships. Cytoscape (http://cytoscape.org/) was used to visualize the PPI network [22, 23]. The hub genes were calculated and identified by using CytoHubba. Molecular Complex Detection (MCODE) plugin was then used to identify gene modules [24]. Enrichment analysis of genes in the top two significant modules was performed by using GO and KEGG.

### Survival analysis

Clinical information of the osteosarcoma cohort with 83 patients was available in the Target database. The patients were divided into two groups according to gene expression value. Kaplan–Meier survival curves were performed using R/Bioconductor (version 3.5.2) [25].

### Gene set enrichment analysis (GSEA)

In the Target cohort, osteosarcoma samples were divided into two groups according to the expression level of two hub genes, respectively. GSEA analysis was performed to identify the potential function of selected hub genes [26]. p value < 0.05 was set as the cutoff to visualize significantly pathways.

## Results

### Immune scores and stromal scores are associated with osteosarcoma clinical status

**Fig. 1:**
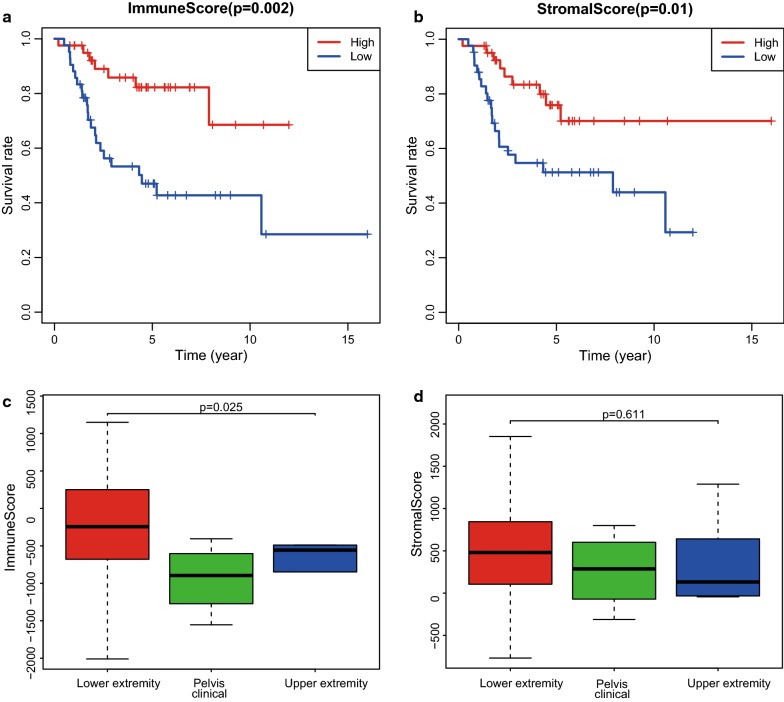
Immune scores and stromal scores are associated with osteosarcoma overall survival and their pathological location. **a** Osteosarcoma cases were divided into two groups based on high and low immune scores. As shown in the Kaplan–Meier survival curve, median overall survival of the high score group is longer than the low score group, as indicated by the log-rank test, p = 0.002. **b** Similarly, osteosarcoma patients were divided into high and low stromal score groups. The median overall survival of the high score group is longer than the low score group by the log-rank test, p = 0.01. **c** Distribution of immune scores of osteosarcoma pathological location. Box-plot shows that there is significant association between osteosarcoma pathological location and the level of immune scores (n = 83, p = 0.025). **d** Distribution of stromal scores of osteosarcoma pathological location. Box-plot shows that there is no significant difference between osteosarcoma pathological location and the level of stromal scores (n = 83, p = 0.611)

We downloaded gene expression profiles and clinical information of all 83 osteosarcoma patients with initial pathologic diagnosis from the Target database. To find out the potential correlation of overall survival with immune scores and/or stromal scores, we divided the 83 osteosarcoma cases into top and bottom halves (high vs. low score groups) based on their scores. Kaplan–Meier survival curves (Fig. 1a) showed that median overall survival of cases with the high score group of immune scores is longer than the cases in the low score group (p = 0.002). Consistently, cases with higher stromal scores also showed longer median overall survival compared to patients with lower stromal scores (Fig. 1b, p = 0.01).

To explore the potential correlation between immune/stromal scores and clinical information, we divided osteosarcoma patients according to tumor location, age, gender, metastasis status and race. Based on the ESTIMATE algorithm, the average immune scores of lower extremity cases ranked the highest of all, followed by upper extremity and pelvis clinical (Fig. 1c, p = 0.025). Similarly, the rank order of stromal scores across pathological location from highest to lowest is lower extremity > pelvis clinical > upper extremity (Fig. 1d, p = 0.611), indicating that immune scores are meaningful in the correlation of pathological location classification. While, there was no statistical difference based on other clinical information classification (Additional file 1: Figure S1).

### Difference of immune cell subsets between high immune score and low immune score groups

**Fig. 2:**
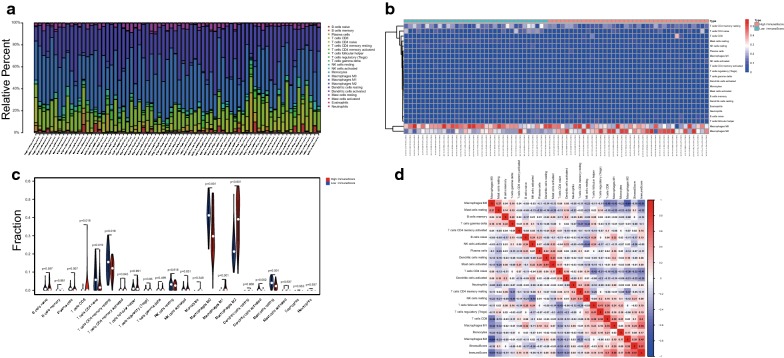
Performance of CIBERSORT across immune cells. **a** The mean proportion of 22 immune cells in the Target dataset. **b** Correlation analysis between high/low immune scores and percentage of 22 immune cells is summarized in the heatmap. **c** Violin plot of high immune score and low immune score groups for the Target cohort, red for high immune score group and blue for low immune score group. The p values showed different infiltration types of immune cells by Wilcox test. **d** Correlation matrix of 21 immune cell proportions and immune/stromal score in the Target datasets. Variables have been ordered by average linkage clustering. For comparison, immune/stromal score has been rescaled to range between zero and one separately in each study

To further analyze the relationship between immune score and immune cells, we used the CIBERSORT algorithm to calculate the percentage of each type of immune cells in osteosarcoma cases (n = 83). When deconvoluted into individual immune cell types, 72 (out of the original 83) samples had CIBERSORT deconvolution p-value less than 0.05. The 22 immune cell proportions of osteosarcoma were observed in Fig. 2a. Macrophages M0, macrophages M2, T cells CD4 memory resting, T cells CD4 naïve, and B cells naïve account for a large proportion of osteosarcoma immune cell infiltration. Figure 2b showed distinct immune cell profiles of cases belong to high immune score (n = 38) vs. low immune score (n = 34) groups. Many differential immune cell types existed between high immune score and low immune score groups (Fig. 2c). Immune score and macrophages M1 showed the strongest positive correlation (Pearson correlation = 0.55), while stromal score and macrophages M2 showed positive correlation (Pearson correlation = 0.28) in the Target database at a CIBERSORT p< 0.05 (Fig. 2d). Of these samples, the least variable immune cell type was eosinophils (0%).

### Analysis of differentially expressed genes

**Fig. 3:**
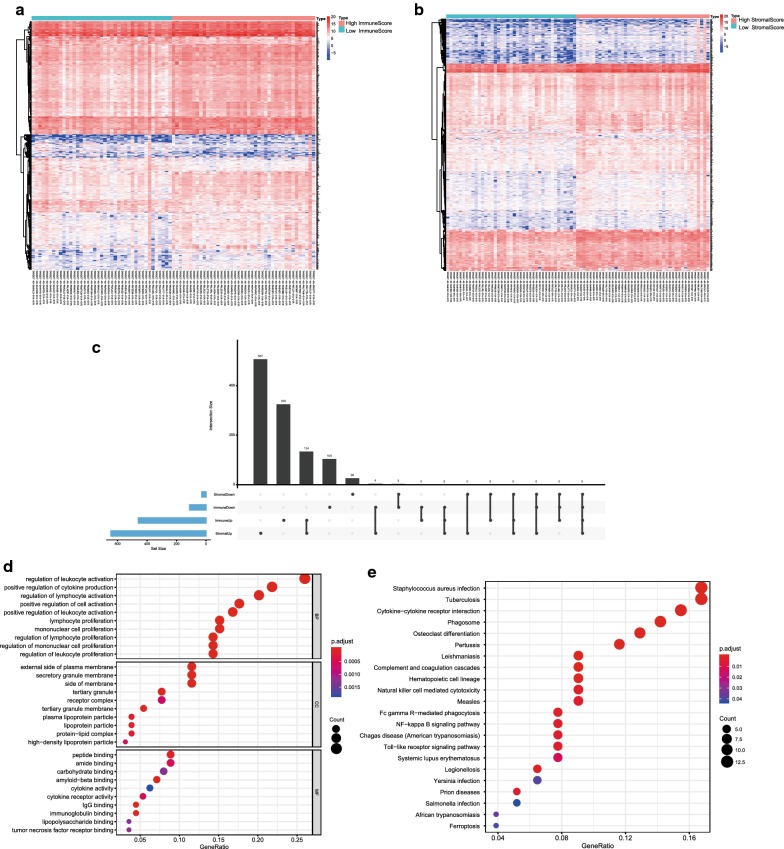
Comparison of gene expression profile with immune and stromal scores of osteosarcoma. **a**, **b** Heatmap of significantly differentially expressed genes based on immune and stromal scores. Red indicates genes with higher expression and blue indicates genes with lower expression. **c** UpSet diagram analysis of aberrantly expressed genes based on immune and stromal scores. **d** Top 30 GO terms was analyzed by clusterProfiler package. **e** KEGG analysis of immune-related pathways

To reveal the correlation of gene expression profiles with immune score and/or stromal score, we compared 83 osteosarcoma cases obtained in the Target database. Heatmaps in Fig. 3a, b showed distinct gene expression profiles of cases belong to high vs. low immune/stromal score groups. For comparison based on immune scores, 459 genes were upregulated and 111 genes were downregulated in the high immune score group than the low score group (fold change > 1.5, p < 0.05). Similarly, based on stromal scores, there were 645 upregulated genes and 29 downregulated genes in the high stromal score group (fold change > 1.5, p < 0.05) (Fig. 3c). Moreover, UpSet plot (Fig. 3c) showed that 134 genes were upregulated in the high score groups, and three genes were downregulated.

To outline the potential function of the DEGs, we performed functional enrichment analysis of the 134 upregulated genes and three downregulated genes (Table 1). GO terms and pathways were indicated by GO and Kyoto Encyclopedia of Genes and Genomes (KEGG) analysis. Top GO terms identified included regulation of leukocyte activation, external side of plasma membrane, and peptide binding (Fig. 3d). The results suggested that these genes were enriched in staphylococcus aureus infection and tuberculosis (Fig. 3e).

**Table 1:** Gene list of 134 up-regulated and three down-regulated genes

Gene	Regulation
CYBB	Up
ZAP70	Up
CD5	Up
FCGR2A	Up
SIRPG	Up
SIGLEC1	Up
LCP2	Up
LTB	Up
PCED1B	Up
APOE	Up
FPR1	Up
TLR4	Up
ITGAM	Up
PLEK	Up
GIMAP8	Up
IL2RA	Up
CD69	Up
SIT1	Up
TIGIT	Up
P2RY6	Up
GJA4	Up
VMO1	Up
FASLG	Up
TNFSF13B	Up
CXorf21	Up
PTPRC	Up
APOC1	Up
CTSS	Up
HPGDS	Up
C1QC	Up
XCL2	Up
CCR5	Up
FCMR	Up
GIMAP1	Up
FPR3	Up
SLAMF8	Up
CSF2RB	Up
CD300LB	Up
SIGLEC7	Up
GIMAP6	Up
MS4A6A	Up
VSIG4	Up
LGALS9	Up
DOCK2	Up
CLECL1	Up
MFNG	Up
FUT7	Up
SIGLEC5	Up
OSM	Up
APOL1	Up
C1QB	Up
CASP1	Up
TLR7	Up
TMC8	Up
C1QA	Up
ADAP2	Up
TMEM150B	Up
C5AR1	Up
RGS18	Up
TFEC	Up
PILRA	Up
CD37	Up
WDFY4	Up
ACSL5	Up
TMIGD3	Up
S1PR4	Up
FCER1G	Up
MS4A4A	Up
CD74	Up
GPR84	Up
C3AR1	Up
LYZ	Up
CD209	Up
PLD4	Up
EBI3	Up
IRF5	Up
IKZF1	Up
LILRB5	Up
ALOX5AP	Up
NPL	Up
CD163	Up
LAIR1	Up
LPAR5	Up
LRRC25	Up
LY86	Up
LTC4S	Up
HMOX1	Up
NRROS	Up
GIMAP5	Up
CETP	Up
ARHGAP30	Up
SH2D1A	Up
CD14	Up
TNFSF8	Up
GPBAR1	Up
MS4A7	Up
SOX17	Up
FGL2	Up
MNDA	Up
CYP2S1	Up
TREM2	Up
MPEG1	Up
CD274	Up
KLRC4-KLRK1	Up
FCGR1A	Up
AGMO	Up
GPR65	Up
SP140	Up
FCGR2B	Up
PTAFR	Up
NFAM1	Up
CECR1	Up
SASH3	Up
CXCR6	Up
FCGR3A	Up
CD200R1	Up
SLCO2B1	Up
FYB	Up
P2RY13	Up
IL10RA	Up
TLR8	Up
C1orf162	Up
C10orf128	Up
LILRA1	Up
TMEM176B	Up
IL1B	Up
SAMSN1	Up
RASGRP4	Up
MSR1	Up
FCGR1B	Up
SIGLEC9	Up
IRF8	Up
GPR34	Up
PDE1B	Up
CSPG5	Down
RPRML	Down
HAND2	Down

### Protein–protein interactions among DEGs

**Fig. 4:**
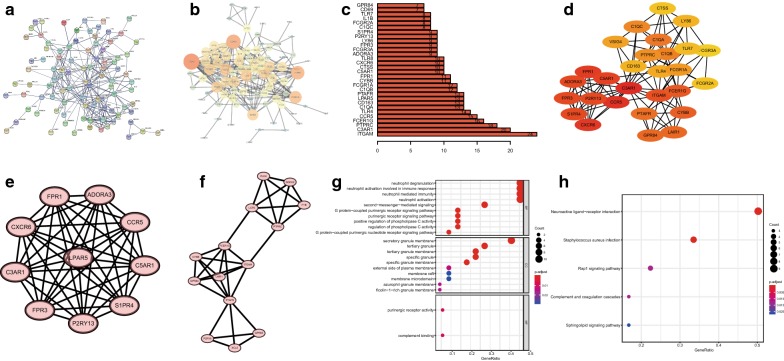
PPI networks of the DEGs and modular analysis. **a**, **b** PPI networks of DEGs using the STRING tool. Genes are represented as nodes in the plot, and their interactions were denoted by lines. The size and color of the nodes represent degree values and change pattern, respectively. The gene of lighter color and greater circle show the higher degree values in this network, whereas the darker color and the smaller circle show the smaller degree values in this network. **c** Bar-plot of top 30 genes with the most weight. **d** Top 30 hub genes using MCC algorithm. The color of a node in the PPI network reflects the p value. **e** Module 1 consists of 10 nodes and 45 edges. **f** Module 2 consists of 14 nodes and 33 edges. GO (**g**) and KEGG (**h**) analysis of genes in the module 1 and module 2. Top five enrichment analysis of GO (include BP, CC, and MF, respectively) and top five pathways KEGG analysis of DEGs. *GO* Gene Ontology, *KEGG* Kyoto Encyclopedia of Genes and Genomes, *BP* biological process, *CC*, cellular component, *MF* molecular function

To better understand the interplay among the identified DEGs, we obtained PPI networks using the STRING tool. The network was made up of five modules, which included 277 intersections and 94 nodes (coincidence interval ≥ 0.7, Fig. 4a). The relationship and function of DEGs were revealed using the PPI network (Fig. 4b). The top 30 genes with the most weight were visualized, which meant they had more connections with other DEGs (Fig. 4c). Figure 4d showed top 30 hub genes based on CytoHubba MCC algorithm by Cytoscape (version 3.7.1). MCODE plugin was used to analyze significant modules in the PPI network, and two modules that had the highest degree were stood out. In the module 1 (Fig. 4e), 45 edges involving 10 nodes were formed in the network. CXCR6, FPR1, ADORA3, CCR5, LPAR5, C3AR1, C5AR1, S1PR4, FPR3, and P2RY13 were the remarkable nodes, as they had the most connections with other members of the module. There were 14 nodes and 33 edges in the module 2 (Fig. 4f). FCER1G, ITGAM, and PFAFR occupied the center of the module. Moreover, genes in the module 1 and module 2 were identified with GO and KEGG analysis. p value < 0.05 and q value < 0.05 were set as the cutoffs to visualize significantly pathways. We found that these genes were mainly involved in neutrophil degranulation and neutrophil activation involved in immune response, and participated in neuroactive ligand–receptor interaction and staphylococcus aureus infection (Fig. 4g, h).

### Validation in the GEO database

**Fig. 5:**
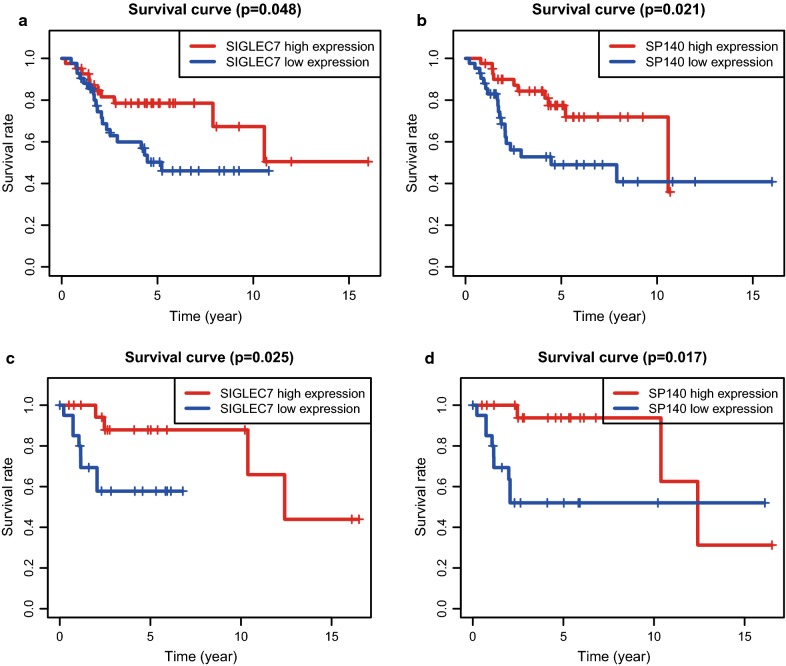
Validation of the Target results in another cohort from the GEO database. Kaplan–Meier survival curves were generated for the verified DEGs associated with overall survival in Target database (**a**, **b**) and GEO database (**c**, **d**). p < 0.05 in log-rank test

To verify that the genes identified from the Target database were also significant in additional osteosarcoma cases, we downloaded and analyzed a cohort of osteosarcoma cases from the GEO database (n = 65), an independent osteosarcoma dataset (Accession number GSE39058). In all, 42 of 65 samples were enrolled for further analysis with complete follow-up data. A total of 70 genes were shown to significantly predict overall survival (Additional file 2: Table S1), of which two genes were associated with better outcomes in osteosarcoma patients (Fig. 5a, b, Target database; c, d, GEO dataset).

### GSEA analysis

**Fig. 6:**
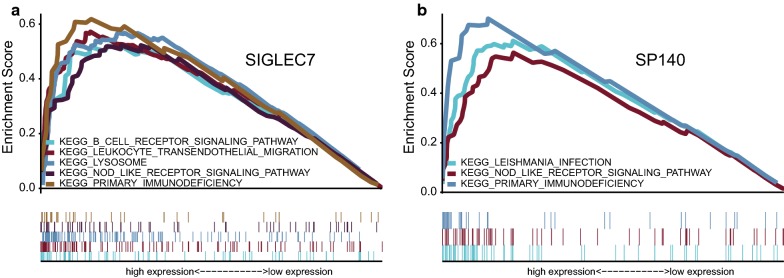
Results of GSEA analysis. Only listed the two prognostic immune-related genes in osteosarcoma samples using GSE39058 cohort

We confirmed that there existed six significant KEGG pathways based on GSEA analysis, including B cell receptor signaling pathway, leukocyte transendothelial migration, lysosome, nod like receptor signaling pathway, primary immunodeficiency, and leishmania infection (Fig. 6).

## Discussion

Osteosarcoma is a rare bone cancer which mainly affects adolescents and young adults. Current standard treatment consists of neoadjuvant chemotherapy, surgical resection of the primary tumor, and adjuvant chemotherapy [27]. Although immunotherapy has heralded much promise for osteosarcoma [28, 29], no biomarkers to stratify patients to distinct therapeutic options currently exist. Moreover, the combination of osteosarcoma genome complexity with the low incidence of these tumors is an obstacle to thorough investigation of osteosarcoma genome biology [30]. Therefore, we sought to identify immune-related prognostic genes that contributed to patients’ overall survival by investigating the TME.

Some reports have applied the ESTIMATE algorithm to several cancers [31–33], demonstrating the effectiveness of the algorithm when applied to expression data. Using the ESTIMATE algorithm, Vincent et al. [31] calculated the purity of breast cancer and demonstrated that differences between tumors and cell lines were attributed to the loss of stromal and immune components in vitro. To understand the microenvironment of osteosarcoma, we utilized the ESTIMATE algorithm to obtain immune and stromal scores. In our study, immune scores were correlated with tumor location of osteosarcoma. In addition, patients with high immune and stromal scores had longer overall survival, suggesting that tumor microenvironment was closely associated with clinical outcomes.

In several studies, the CIBERSORT algorithm was used to examine the relative proportion of infiltrating immune cell subsets in each tumor sample [34–36]. Our work revealed that immune score was positively correlated with macrophage M1, and stromal score was positively associated with macrophages M2. Zhao et al. [35] found that mast cells, natural killer cells, and dendritic cells using CIBERSORT conferred improved distant metastasis-free survival (DMFS), whereas macrophages and T-cells conferred worse DMFS. Whereas, there was no significant correlation between M1-like macrophage or CD8 cell proportion by CIBERSORT with improved DMFS in another study [37]. The results based on CIBERSORT algorithm will need to be compared with other methods such as single-cell RNA sequencing, which might provide a more detailed analysis of the immune cell infiltrates.

Then we divided tumor samples into high and low immune-score and stromal-score groups to identify DEGs. Through GO and KEGG analysis we found that many of them were involved in tumor microenvironment, such as regulation of leukocyte activation, positive regulation of cytokine production, and regulation of lymphocyte activation (Fig. 3). The results were consistent with previous studies that immune cells and extracellular matrix molecules were interrelated in building osteosarcoma microenvironment [38, 39]. Moreover, we constructed protein–protein interaction modules to reveal the relationship and function of DEGs (Fig. 4). Nodes with a high connectivity degree in the modules were related to immune/inflammation response.

Finally, we performed overall survival analysis of 137 DEGs and identified that 70 genes were associated with outcomes in osteosarcoma patients from the Target database. By cross-validation with the GEO dataset, an independent cohort of 42 osteosarcoma cases, we identified two prognostic immune-related genes (Siglec7, SP140) consistent with the Target database. Both genes have not previously been reported in relation to osteosarcoma, indicating potential prognostic biomarkers for further study [40–42].

Depicting a comprehensive landscape of osteosarcoma microenvironment may help to interpret the responses of osteosarcoma to immunotherapies and provide new treatment strategies for patients. The results of our study should be further validated in a prospective cohort of patients receiving immunotherapy. As not all patients have greater benefit from immunotherapy, more clinical factors should be incorporated to construct prediction models.

Several limitations should be considered when interpreting the results. Firstly, owing to the small sample size of the cohort, further verification with big data was necessary. Secondly, due to heterogeneity of osteosarcoma, DEGs identified at the population level may not accurately describe individual tumor sample well. Further experiments were needed to directly determine the specific cell type and proportion of immune infiltration. Thirdly, as the limited availability of osteosarcoma samples, the integration of data from multiple datasets has become a key source of data to study osteosarcoma. However, this method is highly influenced by batch effects due to the lack of statistical controls.

In summary, immune scores and stromal scores calculated based on the ESTIMATE and CIBERSORT algorithms could facilitate the quantification of the immune and stromal components in each tumor sample. Then according to their immune/stromal scores, we categorized osteosarcoma cases in the Target database into high and low score groups, and identified DEGs. Functional enrichment analysis and protein–protein interaction networks further showed that these genes mainly participated in immune/inflammation response. Finally, we validated these genes in an independent osteosarcoma cohort from the GEO database. Thus, we obtained a list of tumor microenvironment-related genes that predicted better prognosis in osteosarcoma patients.

## Conclusions

Our study established an immune-related gene signature to predict overall survival of osteosarcoma, which may help in clinical decision making for targeted molecular therapies.

## Supplementary information

**Additional file 1: Figure S1.** The correlation between the immune/stromal scores and osteosarcoma clinical information. (A) Distribution of immune scores for age < 15/> 25 and 15–25 osteosarcoma cases (p = 0.974). (B) Distribution of stromal scores for age < 15/> 25 and 15–25 osteosarcoma cases (p = 0.837). (C) Distribution of immune scores for female and male osteosarcoma cases (p = 0.147). (D) Distribution of stromal scores for female and male osteosarcoma cases (p = 0.123). (E) Distribution of immune scores for metastatic and non-metastatic osteosarcoma cases (p = 0.218). (F) Distribution of stromal scores for metastatic and non-metastatic osteosarcoma cases (p = 0.203). (G) Distribution of immune scores for Asian, Black or African American, and White osteosarcoma cases (p = 0.416). (H) Distribution of stromal scores for Asian, Black or African American, and White osteosarcoma cases (p = 0.061).

**Additional file 2: Table S1.** Gene list of 70 survival-related DEGs. P value < 0.05 was used as the cut-off.

## Data Availability

The original data of the present study can be found at Target database (https://ocg.cancer.gov/programs/target) and GEO dataset (https://www.ncbi.nlm.nih.gov/geo/).

## Abbreviations

TME: Tumor microenvironment
GEO: Gene Expression Omnibus
DEGs: Differential expressed genes
GO: Gene Ontology
BP: Biological process
MF: Molecular function
CC: Cellular component
FDR: False discovery rate
PPI: Protein–protein interaction
STRING: Search Tool for the Retrieval of Interacting Genes Database
MCODE: Molecular Complex Detection
GSEA: Gene set enrichment analysis
KEGG: Kyoto Encyclopedia of Genes and Genomes
DMFS: Distant metastasis-free survival
