# Supplementary material for: Immune-related prognosis biomarkers associated with osteosarcoma microenvironment
Source: Cancer Cell Int. 2020 Mar 16;20:83. doi: 10.1186/s12935-020-1165-7 (PMC7075043; doi:10.1186/s12935-020-1165-7)
Supplement: Supplementary file 2 — Additional file 2: Table S1. Gene list of 70 survival-related DEGs. P value < 0.05 was used as the cut-off. [file 12935_2020_1165_MOESM2_ESM.pdf]

gene	pvalue
CYBB	0.024473
ZAP70	0.196188
CD5	0.087207
FCGR2A	0.014405
SIRPG	0.034407
SIGLEC1	0.016637
LCP2	0.111605
LTB	0.113191
PCED1B	0.007496
APOE	0.033812
FPR1	0.022448
TLR4	0.016893
ITGAM	0.005056
PLEK	0.013252
GIMAP8	0.210523
IL2RA	0.001085
CD69	0.42859
SIT1	0.036101
TIGIT	0.236315
P2RY6	0.137641
GJA4	0.003738
VMO1	0.372572
FASLG	0.049423
TNFSF13B	0.077027
CXorf21	0.156337
CSPG5	0.030462
PTPRC	0.000229
APOC1	0.002465
CTSS	0.048745
HPGDS	0.011132
C1QC	0.003152
XCL2	0.023277
CCR5	0.011353
FCMR	0.528344
GIMAP1	0.082594
FPR3	0.065309
SLAMF8	0.124111
CSF2RB	0.086825
CD300LB	0.07616
SIGLEC7	0.04809
GIMAP6	0.103417
MS4A6A	0.081689
VSIG4	0.002468
LGALS9	0.013504
DOCK2	0.006829
CLECL1	0.071568
MFNG	0.071208
FUT7	0.081633
SIGLEC5	0.046758
OSM	0.178268
APOL1	0.167673
C1QB	0.015817
CASP1	0.04731
TLR7	0.007836
TMC8	0.110286
C1QA	0.008526
ADAP2	0.083739

TMEM150	0.111522
C5AR1	0.216023
RGS18	0.098694
TFEC	0.01962
PILRA	0.167047
CD37	0.095046
WDFY4	0.029079
ACSL5	0.016034
TMIGD3	0.049975
S1PR4	0.031918
FCER1G	0.000921
MS4A4A	0.001931
CD74	0.059538
GPR84	0.284382
C3AR1	0.014337
LYZ	0.03095
CD209	0.035538
PLD4	0.088151
EBI3	0.008062
IRF5	0.010793
IKZF1	0.020559
LILRB5	0.096548
ALOX5AP	0.067722
NPL	0.135864
CD163	0.00393
LAIR1	0.008016
LPAR5	0.027906
LRRC25	0.106444
LY86	0.041158
LTC4S	0.278368
HMOX1	0.339094
NRROS	0.114746
GIMAP5	0.225698
CETP	0.016834
ARHGAP3	0.032191
SH2D1A	0.205696
CD14	0.013508
TNFSF8	0.011823
GPBAR1	0.224816
MS4A7	0.005943
SOX17	0.198091
FGL2	0.2512
MNDA	0.100821
CYP2S1	0.012448
TREM2	0.044495
MPEG1	0.083345
CD274	0.057809
KLRC4-KL	0.002104
FCGR1A	0.006829
AGMO	0.07368
GPR65	0.025341
SP140	0.020546
FCGR2B	0.025394
PTAFR	0.294658
NFAM1	0.053314
CECR1	0.047926
SASH3	0.122353
RPRML	0.183429

CXCR6	0.128952
FCGR3A	0.104324
CD200R1	0.001975
SLCO2B1	0.000427
FYB	0.856413
P2RY13	0.111397
IL10RA	0.052304
TLR8	0.028739
C1orf162	0.055092
C10orf128	0.013149
LILRA1	0.370273
TMEM176	0.018918
IL1B	0.63451
SAMSN1	0.11463
RASGRP4	0.001901
MSR1	0.04076
HAND2	0.670679
FCGR1B	0.164775
SIGLEC9	0.291845
IRF8	0.075938
GPR34	0.024609
PDE1B	0.005916
